# CRISPR-Cas-Based Diagnostics in Biomedicine: Principles, Applications, and Future Trajectories

**DOI:** 10.3390/bios15100660

**Published:** 2025-10-02

**Authors:** Zhongwu Zhou, Il-Hoon Cho, Ulhas S. Kadam

**Affiliations:** 1 Department of Cardiovascular and Metabolic Sciences, Lerner Research Institute, Cleveland Clinic, Cleveland, OH 44106, USA; 2 Department of Biomedical Laboratory Science, College of Health Science, Eulji University, Seongnam-si 13135, Republic of Korea; 3 Plant Molecular Biology and Biotechnology Research Centre, Division of Applied Life Science, Gyeongsang National University, Jinju-si 52828, Republic of Korea

**Keywords:** CRISPR-Cas, diagnostics, disease, health, biosensor, cancer, SARS-CoV-2, pathogens, Cas9, Cas12a, Cas13a

## Abstract

CRISPR (Clustered Regularly Interspaced Short Palindromic Repeats)-Cas (CRISPR-associated) systems, originally identified as prokaryotic adaptive immune mechanisms, have rapidly evolved into powerful tools for molecular diagnostics. Leveraging their precise nucleic acid targeting capabilities, CRISPR diagnostics offer rapid, sensitive, and specific detection solutions for a wide array of targets. This review delves into the fundamental principles of various Cas proteins (e.g., Cas9, Cas12a, Cas13a) and their distinct mechanisms of action (cis- and trans-cleavage). It highlights the diverse applications spanning infectious disease surveillance, cancer biomarker detection, and genetic disorder screening, emphasizing key advantages such as speed, high sensitivity, specificity, portability, and cost-effectiveness, particularly for point-of-care (POC) testing in resource-limited settings. The report also addresses current challenges, including sensitivity limitations without pre-amplification, specificity issues, and complex sample preparation, while exploring promising future trajectories like the integration of artificial intelligence (AI) and the development of universal diagnostic platforms to enhance clinical translation.

## 1. Introduction

The discovery of Clustered Regularly Interspaced Short Palindromic Repeats (CRISPR) and their associated (Cas) proteins marked a pivotal moment in biotechnology [1,2,3,4]. Originally identified as an adaptive immune system in bacteria and archaea that defends against invading viruses and plasmids, the CRISPR-Cas system has been ingeniously repurposed for various applications, most notably in genetic engineering and, more recently, in molecular diagnostics [5,6,7].

**Figure 1:**
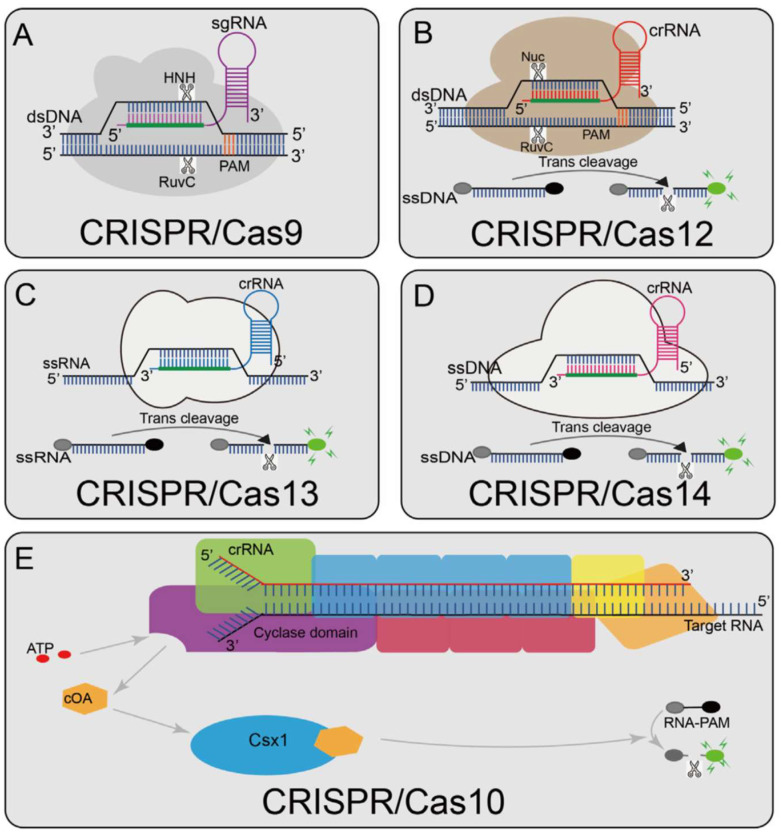
Overview of CRISPR-Cas enzyme activities and their catalytic mechanisms (**A**) Cas9 can cleave the target and non-target strands of DNA; a short trinucleotide PAM is also essential for the initial DNA binding; (**B**) Cas12a can cleave dsDNA under the guidance of gRNA. The Cas12a enzyme recognizes a T-rich PAM and then cleaves the target sequence to generate dsDNA breaks distal to the PAM with staggered 5′ and 3′ ends; Cas12a also has trans-cleavage activity. Once Cas12a binds its target DNA, guided by the crRNA, it unleashes a powerful, non-specific single-stranded DNA (ssDNA) cleavage activity; (**C**) Cas13 activates its single-stranded RNA (ssRNA) cleavage activity by binding to a crRNA, and it has an additional cleavage activity that is triggered by the target RNA; (**D**) Cas14 protein is a RNA-guided nuclease and can recognize the target ssDNA without restriction sequences and cleave it, and also can non-specifically cleave the surrounding ssDNA nucleases molecule; (**E**) Cas10 is a multi-component and multi-pronged immune effector that can be activated by viral RNA, activating Cas10 cyclase activity to produce thousands of cyclic nucleotides (cOA). cOA activates Csx1, cutting off the fluorophore connected to the quencher (Adapted from [12], License: CCBY).

CRISPR diagnostics [7] refers to the application of these bacterial defense mechanisms for the detection of specific DNA or RNA sequences, or other biomarkers, that indicate particular diseases or biological conditions [8,9,10,11]. The core of this technology lies in the programmable nature of Cas enzymes. These enzymes are guided by short RNA sequences, known as CRISPR RNAs (crRNAs) or single guide RNAs (sgRNAs), which direct the Cas proteins to precisely identify and interact with complementary target nucleic acids (Figure 1, [12]). Upon successful binding to the target, the activated Cas enzyme executes a nuclease activity, which is then coupled with a detectable signal, forming the basis of a diagnostic signal [7,11,13].

The emergence of CRISPR diagnostics addresses significant unmet needs in global health and biosensing assay development [14]. Traditional diagnostic methods often face limitations concerning speed, cost, and accessibility, particularly in remote or resource-constrained environments [15]. CRISPR-based solutions offer a compelling alternative by providing rapid, highly sensitive, and specific detection capabilities that can be deployed outside of centralized laboratories. This transformative potential extends to various critical areas, including swift pathogen identification during outbreaks, early detection of complex diseases like cancer, and the advancement of personalized medicine through precise genetic screening.

The rapid transition of CRISPR from a fundamental biological discovery to a versatile diagnostic platform underscores a significant trend in biotechnology: the swift translation of basic scientific understanding into applied technologies [7,16]. This trajectory highlights the power of understanding natural biological mechanisms and repurposing them for human benefit. The inherent precision and programmability of the CRISPR system, which initially demonstrated its prowess in gene editing, directly facilitated its adaptation for diagnostics, establishing it as a revolutionary tool. This progression suggests a broader pattern where bio-inspired engineering drives innovation in diagnostic methodologies (Table 1).

While several excellent reviews have summarized the progress of CRISPR-based diagnostics, the rapid pace of innovation necessitates an updated and comprehensive synthesis. This review aims to fill that gap by not only covering the canonical Cas9, Cas12, and Cas13 systems but also detailing the emerging roles of Cas3, Cas10, and Cas14, providing a broader enzymatic perspective. Furthermore, we provide extensive comparative analyses, benchmarking CRISPR assays against each other and against the gold-standard methods of PCR and Next-Generation Sequencing (NGS). A significant focus is placed on the practical hurdles to widespread adoption, including a detailed discussion of the current regulatory landscape, commercialization efforts, and the path to full clinical integration. Finally, this manuscript offers a forward-looking perspective on the integration of artificial intelligence for assay optimization and the development of universal, “plug-and-play” diagnostic platforms, charting the future trajectories of this transformative technology

## 2. Fundamental Principles of CRISPR-Cas Systems in Diagnostics

The foundational principle of CRISPR diagnostics [5,6,17] is rooted in the remarkable ability of Cas proteins to recognize and cleave specific nucleic acid sequences, a process orchestrated by complementary guide RNAs. This section elaborates on the distinct mechanisms of action employed by key Cas proteins that have been harnessed for diagnostic purposes (Table 2) [14].

### 2.1. Cas9 (Type II)

Cas9 is a widely recognized Cas protein, primarily characterized by its cis-cleavage activity [7,74,75]. In this mechanism, Cas9, guided by a dual RNA (comprising crRNA and tracrRNA, often engineered into a single guide RNA or sgRNA), directly introduces double-strand breaks (DSBs) at specific DNA target sites. This cleavage occurs only when the target DNA sequence is adjacent to a specific Protospacer Adjacent Motif (PAM), a short nucleotide sequence essential for Cas9 binding and activation. Cas9 achieves this precise cutting by utilizing distinct nuclease domains, HNH, and RuvC, each responsible for cleaving one strand of the DNA duplex [5,76,77].

The precision of Cas9’s DNA cleavage makes it fundamental for gene editing applications, which can be adapted for diagnostics. For instance, it can be used to identify specific genetic mutations or the presence of pathogen DNA by generating a detectable signal upon cleavage. Alternatively, a catalytically inactive version of Cas9 (dCas9) can be employed to bind targets without cutting, allowing for visualization or capture of specific nucleic acids [78]. This capability allows for unparalleled precision in directly modifying or detecting specific DNA sequences. This precision is not only crucial for accurate diagnostic identification of genetic variations, such as single-nucleotide polymorphisms (SNPs) or disease-causing mutations (e.g., the BRAF V600E mutation in various cancers) [67,68,79], but also underpins its utility in creating sophisticated disease models. For example, Cas9 has been used to generate knockout mice to study retinal degeneration or rat models for Duchenne muscular dystrophy, thereby indirectly advancing diagnostic capabilities by helping to understand disease pathogenesis and validate new biomarkers [74,75,80].

### 2.2. Cas12a (Type V)

**Figure 2:**
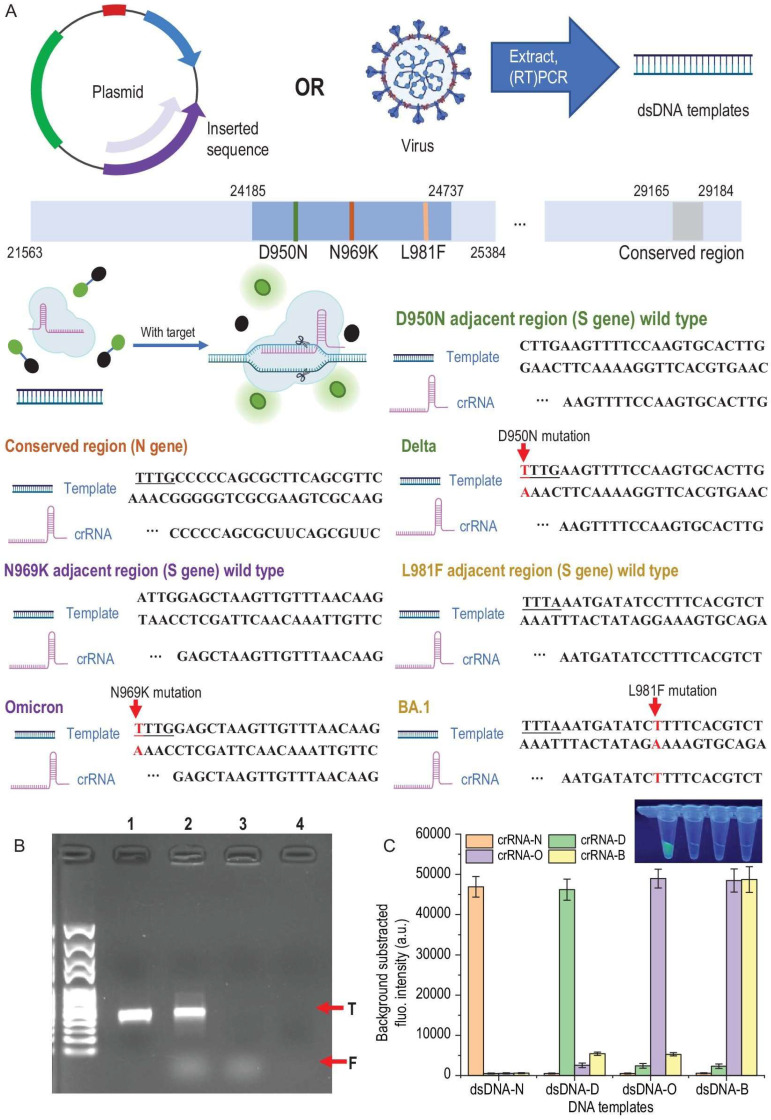
**DNA templates, design of crRNAs and the verification of specificity.** (**A**) dsDNA templates were acquired by amplifying certain inserted sequences in the plasmids or extracting RNA from the virus and performing RT-PCR. SHERLOCK assay was performed by mixing Cas12a protein, crRNA, DNA template and FAM-BHQ probes and incubating at 37 °C for 30 min. Only if the crRNA and DNA were paired would the trans-cleavage happen and freed FAM generate fluorescence. Several mutation sites on S gene sequence and a highly conserved region on N gene sequence were selected as the targets. For D950N and N969K mutation sites, representing Delta and Omicron variants, respectively, TTTV PAM sequences are formed and therefore they have a much higher affinity with the Cas12a protein compared to wild-type sequences. For L981F, which represents the Omicron-BA.1 variant, crRNA was designed according to the mutation sequence. (**B**) Verification of successful trans-cleavage and cis-cleavage was performed using agarose gel electrophoresis. Lane 1, 100 nM DNA template (N gene) only; Lane 2, the 100 nM DNA template performed a SHERLOCK assay, cis-cleavage (T band) and freed FAM coursed by trans-cleavage (F band) were observed; Lane 3, the 1 nM DNA template performed a SHERLOCK assay, although the band of DNA was not observed because of the low loading amount—trans-cleavage (F band) was also observed; Lane 4, 0.01 nM DNA template performed SHERLOCK assay—neither of the bands was observed because the loading amount was too low and no FAM was freed by trans-cleavage. (**C**) Specificity of the crRNA-dsDNA pairs was performed by cross-reactions between each crRNA and dsDNA template (10 nM). Only right paired reactions were observed with strong fluorescence signals: dsDNA-N and crRNA-N; dsDNA-D and crRNA-D; dsDNA-O and crRNA-O; dsDNA-B and crRNA-O/crRNA-B (because BA.1 is one subtype of Omicron). The difference between positive reactions (strong fluorescence) and negative reactions (transparent) is also presented (Adapted from [77], License: CCBY).

**Figure 3:**
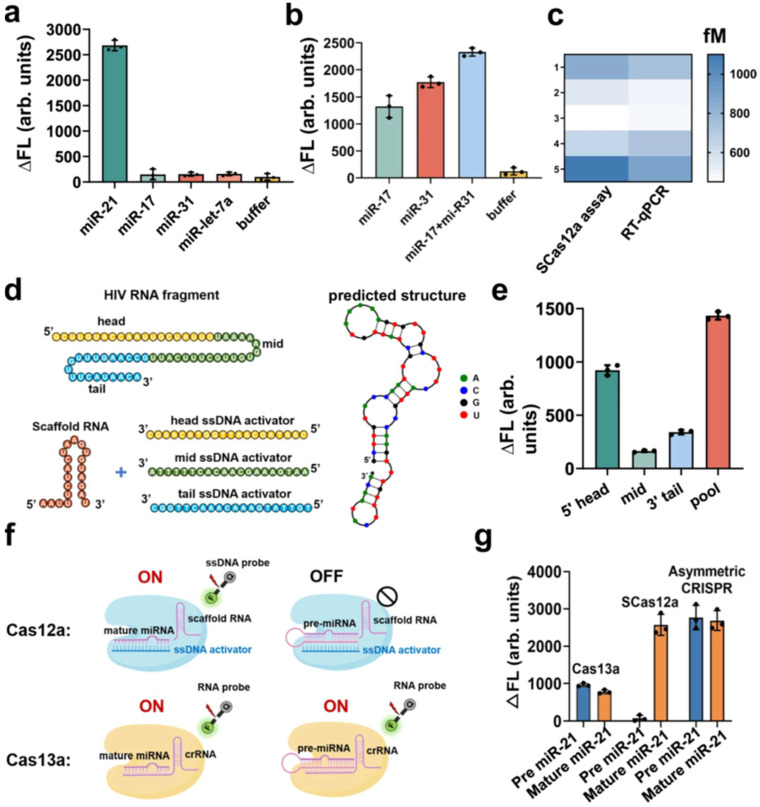
Quantitative detection of RNA by SCas12a assay. (**a**) Selective detection of the target miR-21 (10 pM) in the different types of miRNAs (1 nM), including miR-17, miR-31 and miR-let-7a. (**b**) Multiplexed detection of miR-17 and miR-31 in the same reaction. (**c**) Heatmap of the estimated concentrations of target miR-21 using the SCas12a assay or conventional RT-qPCR assay. (**d**) Schematic of an HIV RNA target and three ssDNA activators targeting it at different positions. The predicted secondary structure of this HIV RNA was determined using NUPACK 4.0 software. (**e**) Comparison among the head, mid, tail and pooled HIV targeting ssDNA activators. (**f**) Schematic of the detection of mature miRNA and pre-miRNA using SCas12a or Cas13a based fluorescence assay. (**g**) Comparison of fluorescence intensity changes between pre miR-21 and mature miR-21 measured using Cas13a, SCas12a or Asymmetric CRISPR assay. The concentrations of pre-miR-21 and mature miR-21 were both 10 nM in each reaction. For (**a**,**b**,**e**,**g**), all the experiments were conducted in triplicate and error bars represent mean value +/− SD (*n* = 3) (Adapted from [87], License: CCBY).

Cas12a, including variants like LbCas12a [81,82] and AsCas12a [83,84], operates through a distinct mechanism known as trans-cleavage, or collateral cleavage [85,86]. Upon recognizing and binding to its specific double-stranded DNA target (a process that also requires a PAM sequence), the Cas12a-crRNA complex undergoes a conformational change that activates its non-specific nuclease activity. This activated Cas12a then indiscriminately cleaves nearby single-stranded DNA (ssDNA) reporter molecules present in the reaction mixture (Figure 2), and it can be used for RNA detection, for example, HIV (Figure 3).

This collateral cleavage activity is highly advantageous for diagnostics because it results in a strong, amplified signal. For instance, if the ssDNA reporter is labeled with a fluorophore and a quencher, its cleavage releases the fluorophore, generating a bright fluorescent signal that is easily detectable. This amplification mechanism makes Cas12a-based assays exceptionally sensitive [87], often integrated with isothermal amplification methods like Recombinase Polymerase Amplification (RPA) or Loop-Mediated Isothermal Amplification (LAMP) to further boost sensitivity for detecting minute quantities of target nucleic acids [48,50,88,89,90,91]. The collateral cleavage mechanism of Cas12a is a cornerstone of its utility in rapid, highly sensitive, and portable diagnostics. This “amplification-by-cleavage” allows for detectable signals from minute quantities of target nucleic acids without relying on complex thermal cycling equipment, a requirement for traditional PCR methods. This directly enables point-of-care (POC) testing in resource-limited settings, as demonstrated by smartphone-based devices for SARS-CoV-2 detection or pen-side tests for animal pathogens [92,93,94].

### 2.3. Cas13a (Type VI)

Cas13a, exemplified by LbuCas13a, is an RNA-guided RNase that specifically targets RNA molecules [95,96]. Similarly to Cas12a, once the Cas13a-crRNA complex binds to its specific RNA target, it becomes activated and exhibits trans-cleavage activity, non-specifically cleaving nearby single-stranded RNA (ssRNA) reporter molecules. Cas13a possesses dual RNase activity: one for crRNA maturation and another for the collateral destruction of target RNA and non-target ssRNA reporters.

**Figure 4:**
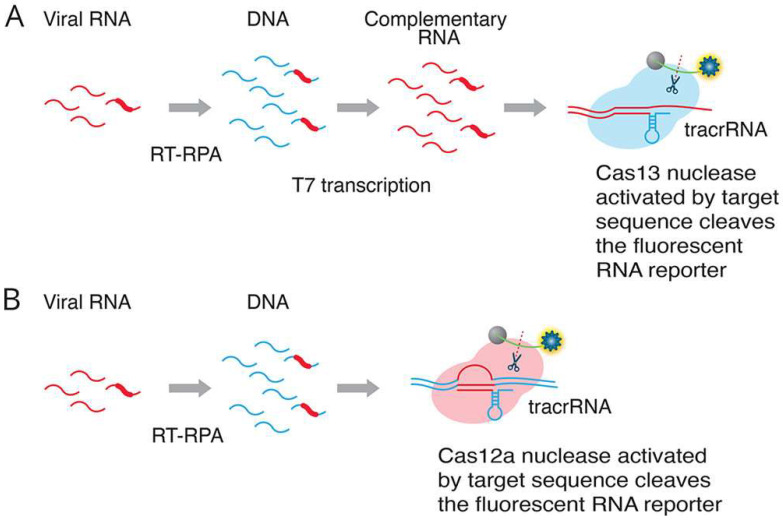
**Two alternative CRISPR methods for detecting viral RNA.** Method (**A**) (SHERLOCK assay [5]: RT-RPA (recombinase polymerase amplification) converts viral RNA to dsDNA. T7 transcription generates complementary RNA from the dsDNA template. The Cas13–tracrRNA complex binds to the target sequence, which activates the general nuclease enzyme activity of Cas13 to cleave the target sequence and the fluorescent RNA reporter. Method (**B**) (DETECTR assay [17]: RT-RPA (recombinase polymerase amplification) converts viral RNA to dsDNA. The Cas12a–tracrRNA complex binds to the target sequence, which activates the general nuclease enzyme activity of Cas12a to cleave the target sequence and the fluorescent RNA reporter (Adapted from [97], License: CCBY).

This collateral RNA cleavage is particularly well-suited for direct detection of viral RNA genomes (e.g., SARS-CoV-2, influenza virus) or RNA biomarkers (e.g., microRNAs) without the need for an initial reverse transcription step, which simplifies workflows and reduces overall turnaround time. Cas13a’s direct RNA targeting and collateral RNA cleavage offer a significant advantage for rapid diagnostics of RNA viruses and RNA biomarkers. This bypasses the need for reverse transcription, a common step in RNA detection, streamlining the workflow and enabling faster results crucial for infectious disease surveillance and outbreak management. For example, the SHERLOCK assay (Figure 4 [97]), a Cas13a-based system, has demonstrated superior sensitivity for early detection of infectious bursal disease virus in chicken tissues [49,96,98,99].

### 2.4. Cas12b (Type V)

Cas12b is another Type V Cas protein that, like Cas12a, mediates trans-cleavage of ssDNA reporters upon target DNA binding [11,100,101]. It also requires a PAM sequence and, in some instances, a tracrRNA for its catalytic activity. Cas12b-based assays are noted for their sensitivity and specificity, often integrated with LAMP for one-pot, one-step detection systems. Examples include the rapid detection of Severe Fever with Thrombocytopenia Syndrome Virus (SFTSV) or Group B Streptococcus (GBS). The growing diversity of Cas enzymes, such as Cas12b, with subtle differences in their PAM requirements, optimal conditions, and collateral activities, provides researchers with a broader toolkit. This allows for the selection and engineering of the most suitable Cas protein for specific diagnostic challenges, leading to optimized assay performance (e.g., improved sensitivity, reduced background) and broader applicability [11,101,102,103,104,105,106,107].

### 2.5. Cas14a (Type V)

Cas14a is a compact Type V Cas protein that also exhibits trans-cleavage activity on ssDNA reporters upon target DNA binding. A notable feature of Cas14a is its PAM-free nature, meaning it does not require a specific PAM for target recognition. This characteristic offers greater flexibility in selecting target sites for diagnostic assay design. Furthermore, Cas14a is highly sensitive to mismatches within its target region, making it particularly valuable for precise single-nucleotide polymorphism (SNP) detection [108,109,110,111,112,113,114]. Cas14a’s PAM-free characteristic represents a significant leap in CRISPR diagnostic design, overcoming a common limitation of many other Cas systems. This, combined with its high sensitivity to single-nucleotide mismatches, enables greater flexibility in targeting diverse genomic regions and enhances the precision required for detecting subtle genetic variations, which is critical for personalized medicine [108,112,115,116,117].

### 2.6. Cas10 (Type III)

Cas10 is a signature protein of Type III CRISPR systems. Its mechanism differs from the direct cleavage observed in other Cas proteins. Upon binding to its target RNA (or DNA in some subtypes), the Cas10 complex initiates the synthesis of cyclic oligoadenylate (cOA) signaling molecules. These cOAs then act as secondary messengers, activating downstream non-specific RNases (e.g., Csm6 or Csx1) that subsequently cleave reporter RNAs, thereby generating a detectable signal [118]. This indirect signaling cascade provides a distinct mechanism for RNA detection, particularly useful for small RNAs, and has been demonstrated in fluorescence and lateral flow assays for parasitic infections [119]. Cas10’s unique mechanism of cyclic oligoadenylate (cOA) synthesis, which then activates a separate reporter cleavage, provides an orthogonal detection pathway compared to direct nuclease activity. This multi-step signaling cascade could potentially offer enhanced robustness and reduced background noise, as the primary recognition event is decoupled from the signal generation step, leading to more reliable diagnostic outcomes [118].

### 2.7. Cas3 (Type I)

Cas3 is a multi-domain nuclease that functions as an integral component of the CASCADE complex in Type I CRISPR systems. This protein possesses both helicase and DNase domains. Upon target DNA recognition by the CASCADE complex (which involves PAM recognition and R-loop formation), Cas3 is recruited and processively degrades the target DNA in a 3′-to-5′ direction [120,121,122]. Its processive degradation offers a distinct approach for diagnostics, potentially enabling quantitative measurements of target load by monitoring the extent of degradation. It is also noted for low off-target effects, which is beneficial for high-fidelity applications. Unlike the single-cut or collateral cleavage mechanisms of other Cas enzymes, Cas3’s processive, unidirectional degradation of target DNA offers a unique advantage for quantitative diagnostics. By monitoring the extent or rate of this degradation, it may be possible to infer the initial concentration of the target nucleic acid with high precision, providing valuable insights into disease burden or treatment response [120,123,124].

## 3. Integrated Technologies Enhancing CRISPR Diagnostics

The utility and versatility of CRISPR diagnostics are significantly amplified through their integration with various complementary technologies. These integrations address critical limitations, particularly concerning sensitivity, sample preparation, and signal readout, thereby expanding the applicability of CRISPR systems beyond the laboratory setting.

### 3.1. Nucleic Acid Amplification

While CRISPR-Cas systems offer high specificity, their inherent sensitivity can sometimes be insufficient for detecting very low concentrations of target nucleic acids present in clinical samples. To overcome this, CRISPR diagnostics are frequently coupled with isothermal nucleic acid amplification methods, which rapidly multiply the target DNA or RNA without requiring complex thermal cycling equipment [125,126,127].

Recombinase Polymerase Amplification (RPA) is a widely adopted isothermal amplification technique [128,129,130]. It operates at a constant, moderate temperature (typically 37–42 °C), eliminating the need for expensive thermocyclers and making it highly suitable for portable and field applications. RPA is frequently coupled with Cas12a or Cas13a systems to achieve ultra-high sensitivity, as seen in assays for infectious bursal disease virus (IBDV), Brucella, and SARS-CoV-2 [38,48,131,132,133].

Loop-Mediated Isothermal Amplification (LAMP) is another robust isothermal amplification technique. LAMP rapidly produces a large amount of DNA from a small amount of target at a constant temperature, making it ideal for simplified, one-pot assays. It is often integrated with CRISPR systems, such as Cas12b, for visual detection assays, as demonstrated in the detection of Monkeypox virus (MPXV) or Group B Streptococcus (GBS) [33,51,52,103,104,134].

Multiple Cross Displacement Amplification (MCDA) is an isothermal amplification method known for its high specificity and sensitivity. It is often used in conjunction with CRISPR-Cas12a for the rapid detection of pathogens like human adenoviruses [135].

The pervasive integration of isothermal amplification methods (RPA, LAMP, MCDA) with CRISPR detection systems reveals a critical symbiotic relationship [104,105,136]. While CRISPR provides unparalleled specificity and collateral activity for signal generation, it often lacks the initial sensitivity required for detecting minute quantities of target nucleic acids, which is a common challenge in clinical samples. Isothermal amplification methods bridge this gap by rapidly multiplying target nucleic acids, making the overall diagnostic assay both highly sensitive and rapid, thereby overcoming a major limitation for clinical utility. This combination allows for rapid, sensitive, and portable diagnostics without the need for sophisticated laboratory infrastructure, directly contributing to the accessibility and cost-effectiveness of these platforms [29,48,88,89,103].

### 3.2. Biosensor Platforms

The signal generated by activated Cas enzymes needs to be translated into a detectable output. Various biosensor platforms are integrated with CRISPR systems to achieve this, offering diverse readout mechanisms and enhancing the analytical capabilities of the diagnostic assays.

Electrochemical Biosensors convert biological recognition events into measurable electrical signals [137,138,139,140]. When integrated with CRISPR-Cas systems, these biosensors offer high sensitivity, miniaturization potential, and compatibility with portable devices, enabling quantitative detection of targets like *Staphylococcus aureus* DNA or viral DNA in extracellular vesicles.

Optical/Fluorescent Biosensors are widely used, leveraging fluorescent reporters that are cleaved by activated Cas enzymes. This cleavage results in a detectable fluorescent signal, allowing for real-time monitoring or endpoint detection using fluorimeters or even simple smartphone cameras. Examples include assays for Group B Streptococcus and human adenoviruses [104,105].

SERS (Surface-Enhanced Raman Spectroscopy) Biosensors combine the specificity of CRISPR with the high sensitivity of Raman spectroscopy for molecular detection [141,142]. These systems offer multiplexing capabilities and ultra-sensitive detection limits, enhancing the analytical power of CRISPR diagnostics.

Integration of Microfluidics and CRISPR-Dx: The microfluidics and CRISPR diagnostics is transforming assays from benchtop prototypes into fast, multiplexed, and truly sample-to-results systems [143,144,145,146,147]. Droplet and chip-based platforms like CARMEN–Cas13 and MiCaR use microfluidic partitioning/coding to pair many guides with many samples, enabling panel-scale detection on a single device, while clinical implementations (e.g., mCARMEN) bring this throughput to variant surveillance. Fully enclosed lab-on-chip designs—such as LOC-CRISPR, IFAST-LAMP-CRISPR, and DISCoVER—string together extraction, isothermal amplification (RPA/LAMP), and Cas12/Cas13 readout to cut hands-on time and minimize contamination. Digital/droplet microfluidics pushes sensitivity and quantitation further, partitioning reactions for absolute copy counting and even amplification-free detection. Physics-assisted chips that co-focus reagents with on-chip electric fields accelerate reaction kinetics, shrinking time-to-result. Field-ready designs (e.g., ALERT) parallelize a handful of reactions for on-site plant and animal testing. Across these threads, microfluidics supplies the throughput, containment, automation, and portability CRISPR needs—turning programmable nucleic-acid sensing into scalable workflows for clinics, farms, food processing lines, and environmental monitoring [145,146,147].

The development of various biosensor platforms integrated with CRISPR systems signifies a strategic effort to diversify readout mechanisms [13,121,141]. This diversification is crucial for expanding the accessibility and applicability of CRISPR diagnostics, catering to different levels of technical infrastructure. From high-throughput laboratory settings that might utilize advanced optical systems to simple, visual, and portable field tests, the choice of biosensor allows for tailored solutions that meet diverse user needs and operational environments. This approach maximizes the potential for widespread adoption and clinical translation [118,148].

### 3.3. Readout Mechanisms

A key aspect of CRISPR diagnostics, particularly for point-of-care applications, is the simplicity and accessibility of result interpretation.

Lateral Flow Assays (LFA) provide a straightforward, visual readout, akin to common pregnancy tests. These paper-based strips are highly portable, require no specialized equipment, and are ideal for rapid point-of-care testing, enabling quick results for infectious diseases like IBDV [48] or *Anaplasma marginale* and *Babesia bigemina* [149,150].

Smartphone Integration represents a significant advancement in portability and data management. Portable devices, often smartphone-based, are designed to provide essential functions such as thermal regulation, fluorescence detection, and automated result interpretation. This integration enables on-site testing, real-time data uploading, and facilitates rapid public health responses during outbreaks.

Some assays are designed to produce a direct Naked-Eye Visual Detection through a color change or visible fluorescence under a simple blue light illuminator. This eliminates the need for complex laboratory instruments and simplifies user interpretation, as demonstrated in assays for *Liposcelis bostrychophila* or prostate cancer screening [151,152].

The strong emphasis on visual and smartphone-integrated readouts is a deliberate strategy to democratize molecular diagnostics. By simplifying the interpretation of results and reducing the reliance on complex laboratory equipment, these methods make advanced testing accessible to non-specialized personnel and in remote, resource-limited environments. This significantly impacts global health surveillance and rapid response capabilities, allowing for timely interventions where traditional laboratory infrastructure is scarce or non-existent.

## 4. Diverse Applications of CRISPR Diagnostics

CRISPR diagnostics have rapidly expanded their utility across a wide spectrum of applications, from infectious disease management to advanced cancer screening and the diagnosis of genetic disorders (Table 3). The programmability and precision of CRISPR-Cas systems enable tailored solutions for diverse diagnostic challenges [153,154,155].

### 4.1. Infectious Diseases

CRISPR diagnostics are extensively applied for the rapid and accurate detection of a wide range of pathogens, which is crucial for effective disease control and prevention, especially in outbreak scenarios.

### 4.2. Bacterial Pathogens

CRISPR-based assays have demonstrated significant promise in identifying various bacterial infections: *Mycobacterium tuberculosis* (TB): Cas12a-based assays, often integrated with RPA, are used for detecting *M. tuberculosis* DNA in blood and respiratory samples. These methods exhibit high sensitivity and specificity, meeting WHO criteria for non-sputum TB diagnostics and are also capable of detecting drug resistance mutations [144,148,156,157,158]. *Staphylococcus aureus*: Amplification-free detection of *S. aureus* DNA is achieved using impedimetric biosensors with CRISPR-Cas proteins [23,24,25,26,27]. Rapid detection of methicillin resistance (*mecA* gene) is also possible using RPA-Cas12a systems [26,28]. *Klebsiella pneumoniae*: Rapid and sensitive detection of *K. pneumoniae*, including carbapenem-resistant strains, is facilitated by one-tube RPA-Cas12a or one-pot Cas12b systems [30,31,32]. *Salmonella* spp.: Various assays, including RPA-Cas12a and LAMP-Cas12b, provide rapid, sensitive, and visual detection of *Salmonella* in both clinical and food samples [159,160]. *Streptococcus pyogenes*: Cas12a systems, combined with amplification and lateral flow, enable rapid, ultrasensitive, and specific detection of *S. pyogenes* [161,162,163]. *Neisseria gonorrhoeae*: Cas13a-based lateral flow assays are being developed for detection in urine and for tracking antibiotic-resistant clones. *Brucella* spp.: Rapid, ultra-sensitive RPA-Cas12a assays have been developed for *Brucella* detection in clinical blood samples, showing strong agreement with qPCR results [38,164]. *Escherichia coli* O157:H7: Label-free and visual detection methods using Cas12a-based biosensors and syringe filters are being developed for food safety monitoring [165,166,167,168,169].

The broad application of CRISPR diagnostics across numerous bacterial pathogens, including those with antibiotic resistance, signifies a shift towards more proactive and precise disease management. Traditional bacterial diagnostics are often slow, relying on culture-based methods, or require extensive laboratory infrastructure, such as with PCR. This can lead to delays in treatment initiation and contribute to the growing problem of antibiotic resistance. CRISPR assays, with their rapid, sensitive, and specific detection capabilities, enable earlier and more appropriate treatment [170]. This reduces the window for disease transmission and minimizes the empirical use of broad-spectrum antibiotics, thereby directly contributing to controlling infectious disease outbreaks and mitigating the escalating threat of antimicrobial resistance, a significant global health concern.

### 4.3. Viral Pathogens

CRISPR diagnostics have proven particularly valuable for rapid viral detection, crucial for public health responses and agricultural biosecurity. SARS-CoV-2 (COVID-19): Rapid, ultrasensitive RPA-assisted Cas12a/Cas13a platforms, often smartphone-enabled, have been developed for self-testing and multiplex detection of respiratory viruses. These systems are also being used for variant detection and wastewater surveillance [171,172]. Human Adenoviruses (HAdV): Rapid, highly specific, and sensitive detection of HAdV is achieved using MCDA-Cas12a or one-pot RPA-Cas13a systems [42]. Human Papillomavirus (HPV): Various Cas12a/Cas9-based biosensors allow for sensitive and amplification-free detection, including genotyping for cervical cancer screening [43,44,45,173]. Infectious Bursal Disease Virus (IBDV): The SHERLOCK (Cas13a-based) assay provides ultrasensitive detection in poultry, which is critical for early disease management in the agricultural sector [48,49]. Monkeypox Virus (MPXV): One-step LAMP-Cas12b assays offer rapid, sensitive, and visual detection, making them suitable for resource-limited settings [51,52,174]. Dengue Virus (DENV): Rapid RAA-Cas13a assays enable serotype-specific detection in a single-tube format, enhancing diagnostic efficiency [175,176]. Influenza Virus: Multiplex detection with SARS-CoV-2 is possible using Cas13a immunochromatographic strips [121,177,178].

The extensive development of CRISPR diagnostics for a wide array of viral pathogens, ranging from human respiratory viruses (e.g., SARS-CoV-2, influenza) to agricultural threats (e.g., IBDV), highlights its critical role in pandemic preparedness and biosecurity. Viral outbreaks, such as the COVID-19 pandemic, underscore the urgent need for rapid and widespread testing, a capability often lacking in traditional diagnostic methods [121,153,155,177,178]. CRISPR’s speed, portability, and visual readout capabilities enable swift identification of infection sources and timely interventions, which are crucial for controlling large-scale outbreaks. Its application in animal health also demonstrates its utility in agricultural biosecurity, helping to minimize economic losses. The ability to detect specific variants further enhances surveillance capabilities, establishing CRISPR as a key tool for global health and food security.

### 4.4. Parasitic Infections

CRISPR systems are also being adapted for the diagnosis of parasitic diseases, particularly in contexts where traditional methods are limited. *Trypanosoma cruzi*: Cas12a-based systems are employed for rapid screening of *T. cruzi* in Chagas vectors and reservoirs [179,180,181]. *Toxoplasma gondii*: Novel LAMP-Cas12b methods facilitate rapid and visual detection of *T. gondii* in environmental samples [58,59,182]. *Anaplasma marginale* and *Babesia bigemina*: CRISPR-Cas12a-based pen-side diagnostic tests have been developed for these tick-borne pathogens in cattle, offering sensitive and user-friendly solutions for livestock health [149,150].

The application of CRISPR diagnostics to parasitic infections, particularly in animal health and zoonotic diseases, demonstrates its potential to address diagnostic gaps in often-neglected areas. Diagnosis of parasitic diseases, especially in livestock or remote regions, frequently relies on less sensitive methods like microscopy or necessitates complex laboratory equipment. CRISPR-Cas systems are being developed into sensitive and user-friendly “pen-side” tests or rapid visual detection methods, making advanced molecular diagnostics accessible for diseases like Anaplasmosis and Babesiosis in cattle, and zoonotic infections like Toxoplasmosis. This directly addresses the need for diagnostics in resource-limited settings and for diseases that might not receive as much attention as major human epidemics, thereby improving both animal health and public health outcomes.

### 4.5. Food, Agriculture and Environmental Monitoring

Beyond human and animal health, CRISPR diagnostics contribute to food security and agricultural sustainability. CRISPR-Cas12a has been successfully used for the rapid and amplification-free detection of plant pathogens such as Tomato Leaf Curl Karnataka Virus (ToLCKV) [61] and *Pantoea stewartii* [183]. The technology is also employed for the detection and verification of genome-edited plants [184].

CRISPR tests can rapidly screen foods for pathogens like *Salmonella*, *E. coli*, and *Listeria*—often in under an hour at plants or import checkpoints—avoiding days-long culture/PCR workflows. Portable assays (e.g., Cas13 on lettuce wash water) flag contamination early, and programmable guides enable quick checks of species identity to detect food fraud or mislabeling [25,77,109,185].

On-site CRISPR diagnostics let farmers and vets detect crop and livestock pathogens (e.g., citrus greening, African swine fever, avian influenza) within an hour for targeted containment. They also verify GM traits by detecting specific DNA signatures. Emerging multiplex formats can screen several pathogens or traits in a single test, reducing the need for multiple PCRs. CRISPR enables field-ready detection of pathogens or contaminants in water (including wastewater surveillance), and biosensor formats are being adapted for certain toxins or metals. eDNA workflows use CRISPR to spot trace DNA from endangered or invasive species, while routine monitoring can extend from bioreactors to beaches (e.g., harmful algal blooms) [139,140,186,187,188].

The extension of CRISPR diagnostics to plant and agricultural health signifies its role beyond human medicine, directly contributing to food security and sustainable agriculture. Plant pathogens pose significant economic threats and require quick, accurate detection for effective containment. Rapid and accurate detection of these pathogens enables early intervention, reducing crop losses and minimizing the need for broad-spectrum pesticides [14,189,190,191]. This promotes more efficient and environmentally friendly farming practices, ultimately enhancing global food security.

### 4.6. Cancer Diagnostics

**Figure 5:**
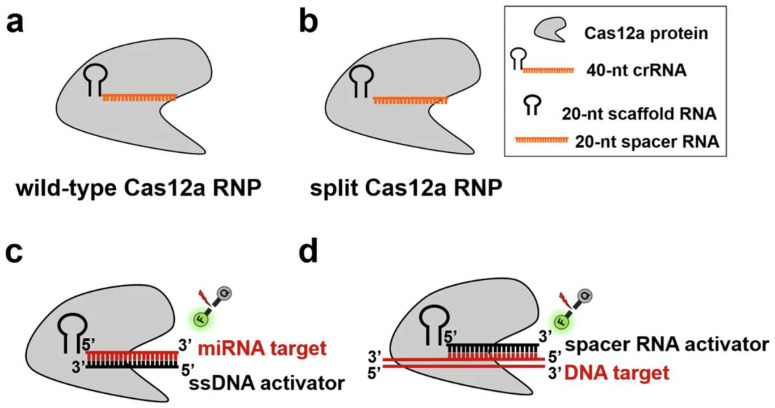
Schematic illustration of the split Cas12a-based assay for the direct detection of miRNA and DNA. (**a**) Schematic illustration of the wild-type Cas12a RNP. (**b**) Schematic illustration of the split Cas12a RNP. (**c**) The principle of the amplification-free fluorescence assay developed for the direct detection of miRNA target in this study. (**d**) The principle of the fluorescence assay for the direct of DNA target in this study (Adapted from [87], License: CCBY).

CRISPR diagnostics are emerging as powerful tools for early cancer detection, prognosis, and guiding personalized treatment strategies by identifying specific molecular biomarkers. MicroRNAs (miRNAs)—Highly sensitive detection of cancer-associated miRNAs is achieved using Cas12a/Cas13a systems. Examples include miR-196a2 for general cancer diagnosis, hsa_circ_0049101 for ovarian cancer, and multiplex detection of various miRNAs (Figure 5 [87]) or for colorectal cancer detection [62,63,64,65,66]. Single-Nucleotide Polymorphisms (SNPs)—Precise genotyping of cancer-associated SNPs, such as the BRAF V600E mutation, is performed using RPA-Cas12a or Cas12a/Cas13a systems. This is crucial for selecting optimal therapeutic strategies [5,67,68]. Extracellular Vesicle (EV)-bound DNA/RNA—The technology enables the detection of viral DNA in circulating extracellular vesicles for monitoring oncovirus-related disease progression. It also facilitates the detection of EV-bound S100A8/A9 for predicting septic shock and acute respiratory distress syndrome (ARDS) [186,192,193,194]. Specific Cancer Biomarkers—Detection of Prostate Cancer Associated 3 (PCA3) [69] in urine offers a non-invasive method for prostate cancer screening, potentially appearing earlier than traditional markers like prostate-specific antigen (PSA). Additionally, CRISPR systems aid in the identification of hub mRNAs and lncRNAs implicated in colorectal cancer pathogenesis [195,196].

The application of CRISPR diagnostics in cancer, particularly for detecting subtle molecular changes like miRNAs, SNPs, and EV-bound nucleic acids, marks a significant advance towards precision oncology. Early and precise cancer diagnosis is often challenging, relying on invasive biopsies or less specific biomarkers. CRISPR diagnostics offer high sensitivity and specificity for detecting these molecular fingerprints of cancer, enabling non-invasive “liquid biopsies” for early detection and monitoring disease progression. This capability allows for more accurate risk stratification and personalized treatment selection, moving beyond traditional, often invasive, diagnostic methods and contributing significantly to tailored cancer therapies [197,198,199].

### 4.7. Genetic Disorders

CRISPR systems are also leveraged for identifying genetic mutations linked to inherited diseases and for creating disease models to advance therapeutic understanding. Mutation Detection—CRISPR-based assays can detect specific mutations, such as the BRAF V600E mutation or novel variants in genes like FBN1 for Marfan syndrome [5,67,68]. Disease Modeling—CRISPR/Cas9 is extensively used to generate accurate animal models (e.g., rat models for Duchenne muscular dystrophy, mouse models for galactosemia or congenital hydrocephalus) [71]. These models are invaluable for studying disease mechanisms, elucidating underlying molecular pathways, and testing the efficacy of novel therapeutic interventions, including gene therapies [200].

The use of CRISPR in diagnosing and modeling genetic disorders highlights its dual role: precise identification of pathogenic mutations and facilitating the understanding and development of gene therapies. Genetic disorders are caused by specific gene mutations, requiring precise detection and often lacking effective treatments. CRISPR-based assays can detect these mutations with high accuracy and resolution. Furthermore, CRISPR/Cas9 is used to create animal models that faithfully mimic human genetic conditions. These models are critical for elucidating disease mechanisms and testing gene-editing or other therapeutic strategies. Thus, CRISPR not only diagnoses but also directly informs and accelerates the development of treatments for genetic diseases, paving the way for a future of personalized genetic medicine [201,202,203].

## 5. Key Advantages of CRISPR-Based Diagnostic Assays

**Figure 6:**
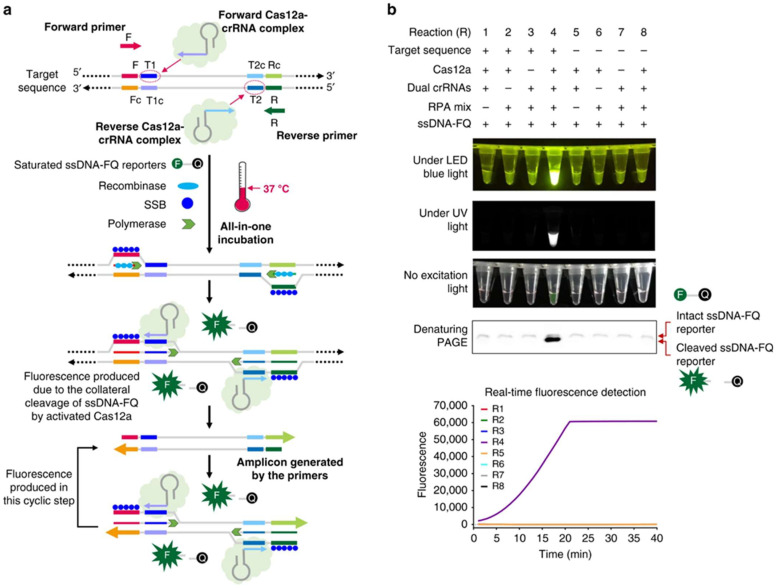
**Design and working principle of the AIOD-CRISPR assay.** (**a**) Schematic of the AIOD-CRISPR assay system. SSB is single-stranded DNA binding protein. The four sites in the target sequence are labeled as F, T1, T2, and R, respectively. The letter c represents the corresponding complementary site. For example, F and Fc sites are complementary. The short horizontal lines with the same colors denote the same sites and their arrows represent the direction of 5′–3′. (**b**) Evaluation of eight AIOD-CRISPR reactions (R) with various components through endpoint imaging after 40 min incubation, denaturing polyacrylamide gel electrophoresis (PAGE) analysis of the single-stranded fluorescent reporter (ssDNA-FQ), and real-time fluorescence detection. The ssDNA-FQ was labeled by 5′ 6-FAM (Fluorescein) fluorophore and 3′ Iowa Black FQ quencher. Recombinase polymerase amplification (RPA) mix from TwistAmp Liquid Basic kit was composed of 1× Reaction Buffer, 1× Basic E-mix, 1× Core Reaction Buffer, 14 mM MgOAc, 320 nM each of primers, and 1.2 mM dNTPs. Dual crRNAs contained 0.64 μM each of crRNAs specific to the SARS-CoV-2 N gene sequence. A plasmid containing the SARS-CoV-2 N gene sequence (3 × 10^3^ copies), 8 μM of ssDNA-FQ reporters, and 1.28 μM EnGen Lba Cas12a (Cpf1) were used. Each experiment was repeated three times with similar results (Adapted from [178], License: CCBY).

CRISPR-based diagnostic assays (Figure 6, [204]) offer a compelling suite of advantages that position them as a transformative technology in molecular diagnostics (Table 3). These benefits collectively address many limitations of conventional diagnostic methods, particularly for point-of-care (POC) applications and use in resource-limited settings.

### 5.1. Speed and Rapid Turnaround Times

A hallmark of CRISPR diagnostics is their remarkable speed. Many assays significantly reduce the time from sample collection to result, often delivering outcomes within 15 min to an hour, a stark contrast to conventional methods that can take several hours or even days. For instance, the HAdV-MCDA-CRISPR assay provides results in approximately one hour, including template preparation, amplification, and interpretation [42]. The PalmCS platform for Group B Streptococcus (GBS) screening offers a “sample-in-result-out” time of just 20 min [205]. This rapid feedback is critical for timely clinical decisions, enabling immediate patient management, and for swift public health responses during infectious disease outbreaks [203,206].

### 5.2. High Sensitivity and Specificity

CRISPR diagnostics are renowned for their analytical performance. These assays can detect extremely low concentrations of target nucleic acids, frequently achieving attomolar (aM) or even single-copy detection limits. For example, the IBD-SHERLOCK assay boasts a detection limit of 5 aM, equivalent to just three IBDV-RNA molecules [48,49]. Their programmability, guided by precise crRNA-target complementarity, ensures high specificity, enabling the differentiation of closely related sequences, including single-base mismatches. This precision is crucial for accurate pathogen identification, distinguishing between viral strains, detecting specific genetic mutations, and identifying cancer biomarkers. The PalmCS system for GBS, for instance, demonstrated 97.5% sensitivity and 100% specificity in clinical samples [207,208].

### 5.3. Portability and Suitability for Point-of-Care (POC) Testing

Many CRISPR diagnostic platforms are intentionally designed to be compact, lightweight, and operable with minimal equipment, making them ideal for decentralized testing outside of traditional laboratory settings. This includes palm-sized devices and systems that integrate with smartphones for detection and interpretation. The ability to perform tests at the point of care, such as in chicken farms for IBDV detection or in remote settings for Monkeypox virus, significantly improves accessibility and enables rapid on-site decision-making [209,210,211].

### 5.4. Cost-Effectiveness and Reduced Equipment Requirements

By simplifying workflows, reducing reagent volumes, and often eliminating the need for expensive thermocyclers or complex optical instruments, CRISPR diagnostics offer a more affordable solution compared to traditional molecular tests. Research efforts are focused on developing “room temperature CRISPR diagnostics” to further reduce complexity and equipment requirements, making them particularly beneficial for low- and middle-income countries. The cost of reagents, such as heparin sodium in some assays, can be as low as USD 0.01-USD 0.04 per thousand uses, highlighting the potential for significant cost reduction [5,209,211,212,213].

### 5.5. Visual Detection Capabilities

A significant practical advantage of many CRISPR assays is the ability to interpret results visually. This is often achieved through a clear color change or fluorescence visible under a simple blue light illuminator, removing the need for sophisticated detection instruments and simplifying user interpretation. Examples include naked-eye visualization for MPXV detection or colorimetric readouts for prostate cancer screening. This feature is particularly valuable for self-testing and field applications where laboratory infrastructure is unavailable [45,214,215,216,217,218].

### 5.6. Multiplexing and Versatility

Some advanced CRISPR platforms possess the capability to simultaneously detect multiple targets (multiplexing) from a single sample, providing comprehensive diagnostic information and potentially reducing overall analysis costs. For instance, multiplex detection of respiratory RNA viruses like SARS-CoV-2 and influenza has been demonstrated. The inherent programmability of guide RNAs makes the entire technology highly versatile, allowing for the detection of a diverse range of DNA and RNA targets, from pathogens to cancer biomarkers and genetic mutations [209,219,220].

### 5.7. Reduced Contamination Risk

Many CRISPR assays are designed as “one-pot” or “closed-tube” systems, which minimize sample handling and significantly reduce the risk of aerosol contamination. This is a common source of false positives in traditional nucleic acid amplification tests, and its reduction enhances the reliability of CRISPR diagnostics. The integrated tube design in platforms like PalmCS [104], which combines nucleic acid extraction, gene amplification, and CRISPR reaction, exemplifies this advantage [221,222].

### 5.8. Amplification-Free Detection

While many CRISPR assays benefit from pre-amplification, certain advanced systems can detect nucleic acids directly without this step. This further simplifies the workflow, reduces turnaround time, and minimizes contamination risks. Examples include impedimetric biosensors for *Staphylococcus aureus* DNA and some CRISPR CLAMP systems for microRNAs [223,224,225,226].

The collective advantages of CRISPR diagnostics—speed, high sensitivity, specificity, portability, cost-effectiveness, and visual readout—represent a powerful convergence that fundamentally redefines the accessibility of molecular diagnostics [155]. This is not merely an incremental improvement but a paradigm shift that enables sophisticated testing to move from centralized, high-resource laboratories to decentralized, low-resource settings. This empowers rapid, on-site decision-making in public health, clinical care, and even agriculture. By combining high analytical power with practical accessibility, CRISPR diagnostics are poised to address critical global health disparities and enable swift responses to emerging health crises or the early detection of chronic diseases.

## 6. Challenges and Limitations in CRISPR Diagnostics

Despite the significant promise and numerous advantages of CRISPR diagnostics, several challenges and limitations must be addressed for their widespread adoption and full clinical integration (Table 4). These hurdles span technical aspects of assay performance, practical considerations in sample processing, and the complex landscape of clinical implementation and regulation.

### 6.1. Achieving Consistent Ultra-High Sensitivity Without Preamplification

While some CRISPR assays claim to achieve amplification-free detection, many, particularly those utilizing Cas12a, still necessitate a pre-amplification step (e.g., LAMP, RPA) to attain the ultra-high sensitivity required for detecting very low target concentrations in complex clinical samples. The reliance on an initial amplification step, while effective, adds a layer of complexity to the overall workflow and can reintroduce some of the contamination risks that CRISPR-based systems aim to mitigate. Developing robust amplification-free methods that consistently meet clinical sensitivity benchmarks across diverse sample types remains an active area of research [153,227].

### 6.2. Specificity Issues

While CRISPR systems are generally praised for their high specificity, certain scenarios can pose challenges, such as, off-target effects: Despite careful design, CRISPR systems can occasionally exhibit off-target activity, binding to or cleaving sequences that are not the intended target. This can lead to false positive results or unintended consequences in diagnostic applications [228]. Cross-reactivity: In multiplex assays or when dealing with highly homologous pathogens, designing crRNAs that ensure absolute specificity can be challenging. Cross-reactivity with closely related pathogens or homologous sequences may occur, potentially leading to false positives or ambiguous results [229,230]. Homologous RNA interference: For RNA-targeting systems like Cas13a, the presence of homologous linear RNAs can interfere with the precise detection of specific RNA targets, such as circular RNAs, posing a significant obstacle for their application in molecular diagnostics [145,187,231,232,233,234].

### 6.3. PAM Dependency

A notable limitation for many commonly used Cas enzymes, including Cas9 and Cas12a, is their requirement for a specific PAM sequence located adjacent to the target DNA. This dependency restricts the available target sites within a genome, which can be a constraint for designing assays for certain genomic regions or for detecting all possible mutations within a target sequence. While some newer Cas variants like Cas14a are PAM-free, their broader adoption and integration into diverse diagnostic platforms are still evolving [146,235,236].

### 6.4. Background Noise and False Positives

Non-specific collateral cleavage or other unintended enzymatic activities can contribute to background noise and lead to false positive signals. This issue is particularly pertinent in highly sensitive assays or when analyzing complex sample matrices that may contain interfering substances. Minimizing such background activity while maintaining high sensitivity is a continuous challenge in the optimization of CRISPR diagnostic assays [237,238,239].

### 6.5. Complex Sample Preparation Requirements

Despite the inherent simplicity of the CRISPR detection step itself, many assays still require upstream sample preparation steps. These steps, such as nucleic acid extraction and purification, can be labor-intensive, time-consuming, and often necessitate specialized equipment. This requirement significantly hinders the realization of a true “sample-in-result-out” point-of-care functionality, especially in low-resource settings where such infrastructure is limited [240,241,242,243].

### 6.6. Clinical Implementation Hurdles and Regulatory Pathways

Transitioning CRISPR diagnostics from research laboratories to widespread clinical and commercial use involves substantial non-technical hurdles [244,245,246,247]. Validation and standardization—Extensive clinical validation across diverse patient cohorts is essential to ensure the robustness, reliability, and reproducibility of diagnostic results. Furthermore, standardization of protocols, reagents, and quality control measures is crucial for widespread adoption and comparability across different settings. Regulatory Approval—Navigating the complex regulatory pathways for diagnostic devices and assays is a significant and often lengthy process. Demonstrating safety, efficacy, and clinical utility to regulatory bodies is a major hurdle for commercialization and clinical deployment [7,248]. Manufacturing and Scalability—Producing high-quality, stable Cas proteins and guide RNAs at scale and in a cost-effective manner remains a challenge for widespread commercialization. Ensuring consistent batch-to-batch quality is paramount for reliable diagnostic performance.

The identified challenges reveal a critical tension in diagnostic innovation: the trade-offs between achieving ultra-high analytical performance (sensitivity, specificity) and ensuring practical, user-friendly, and cost-effective clinical implementation. Overcoming these limitations requires not just further enzyme engineering but also holistic assay design that considers the entire workflow, from sample collection to result interpretation. This approach must address the complex interplay of biological, technical, and logistical factors to ensure real-world utility. The development of robust solutions that balance cutting-edge scientific capabilities with practical usability and scalability is essential for CRISPR diagnostics to fulfill their transformative potential [5,249,250].

## 7. Future Perspectives and Emerging Trends

The field of CRISPR diagnostics is dynamic, with continuous innovation aimed at overcoming current limitations and expanding its capabilities. Key future perspectives and emerging trends revolve around the integration of advanced computational tools, the development of highly versatile platforms, and a concerted effort towards seamless clinical translation [155,188,251].

### 7.1. AI Integration

Artificial intelligence (AI), particularly machine learning and deep learning, is poised to revolutionize CRISPR diagnostics by enhancing various aspects of assay development, optimization, and interpretation. Assay optimization and design—AI algorithms can optimize crRNA design to target highly conserved viral regions, thereby enhancing both sensitivity and specificity. They can also predict sgRNA activity for gene-editing applications that inform diagnostics, leading to more efficient and reliable assay components. Automated data analysis and interpretation—Machine learning algorithms are increasingly being developed to automate the interpretation of complex diagnostic signals. This includes analyzing fluorescence images from digital CRISPR devices and interpreting results from lateral flow assays, which significantly reduces human error and improves diagnostic speed and accuracy. Biomarker discovery and risk stratification—AI-powered analyses of multi-omics data, such as RNA sequencing and genome-wide knockout screens, can identify novel biomarkers for diseases like colorectal cancer and preeclampsia. These analyses can also build predictive models for risk stratification and prognosis, moving towards more personalized diagnostic and treatment strategies [19,20,21,22,252,253,254].

The pervasive integration of AI into CRISPR diagnostics signals the emergence of an intelligent diagnostic ecosystem. This goes beyond simply automating tasks; AI will enable predictive capabilities for assay design, real-time adaptive optimization during testing, and sophisticated interpretation of complex biological data. For example, AI can predict optimal crRNA sequences, interpret intricate fluorescence patterns beyond human visual capacity, and identify novel biomarkers from vast datasets. This fundamentally transforms how diagnostics are developed, performed, and utilized for personalized medicine and public health surveillance. This suggests a future where AI is not merely a tool within a CRISPR assay but an integral part of the entire diagnostic lifecycle, from initial research and development to real-time clinical application and public health monitoring, leading to more adaptive, accurate, and autonomous diagnostic solutions [253,254,255].

### 7.2. Universal CRISPR Platforms

A key future direction involves the development of universal CRISPR platforms that are highly adaptable and programmable, capable of detecting a wide range of targets without requiring significant re-engineering for each new analyte. Modular design--innovative strategies, such as modular electrochemical sensing, fragment complementary activation, and split crRNA designs, aim to create versatile core systems. In these systems, only the crRNA needs to be swapped for different targets, drastically simplifying the development process for new assays [185,256,257]. Amplification-free and one-tube systems: Continued efforts are focused on developing robust amplification-free detection methods and integrated one-tube/one-pot assays. These designs minimize complexity, reduce contamination risk, and lower equipment needs, making the assays more accessible and user-friendly. Broad applicability: These universal platforms are designed to be applicable across diverse sample types and for various analytes, including DNA, RNA, microRNAs, exosomes, and even proteins. This broad utility enhances their impact in human health, food safety, and environmental monitoring [257,258].

The push for “universal CRISPR platforms” signifies a strategic move towards a “plug-and-play” model for molecular diagnostics. By creating highly modular and adaptable core systems, researchers aim to drastically reduce the development time and cost for new assays. This enables rapid deployment against emerging threats or for new biomarker discoveries, thereby enhancing diagnostic agility. For instance, during a new pandemic, instead of developing a completely novel assay from scratch, an existing universal platform could be rapidly reconfigured by simply changing the guide RNA. This greatly enhances diagnostic responsiveness to unforeseen health challenges, making the technology truly scalable for global needs [259,260,261,262].

### 7.3. Enhanced Clinical Translation, Commercialization, and Regulatory Aspects

Future efforts will increasingly focus on transitioning CRISPR diagnostics from academic research laboratories to widespread clinical and commercial use. A primary focus will be on developing assays that are robust and perform reliably in real-world clinical samples, minimizing the need for complex sample preparation, and are designed to be user-friendly for non-expert operators. Exploration of sustainable diagnostic solutions that are both environmentally friendly and economically viable for long-term, global implementation will be critical. Establishing clear regulatory guidelines and streamlined pathways will be essential to facilitate the approval and market entry of CRISPR-based diagnostic products, ensuring their safety and efficacy for public use [46,147,263].

A few companies (Sherlock Biosciences, Mammoth Biosciences, CASPR Biotech, etc.) have been spearheading the commercialization of CRISPR diagnostics. During the COVID-19 emergency, some CRISPR tests achieved regulatory authorizations: for example, Mammoth’s SARS-CoV-2 DETECTR kit and Sherlock’s SARS-CoV-2 SHERLOCK kit both received FDA Emergency Use Authorization (EUA) in the U.S. These EUAs demonstrated that CRISPR diagnostics can meet regulatory standards for accuracy and can be scaled during a public health crisis. However, EUAs are temporary; as of 2025, no CRISPR assay has full FDA approval for routine clinical use beyond emergency or research contexts. The Sherlock COVID-19 test, for instance, was authorized only for use in high-complexity CLIA laboratories (not as an over-the-counter test) due to its multi-step protocol. This underscores the gap between impressive lab results and practical, regulatory-cleared products [9,10,17,49,97].

Looking ahead, CRISPR diagnostics will likely need to undergo the FDA’s in vitro diagnostic (IVD) approval process (or similar in other countries) for each intended use. This entails robust clinical validation, quality manufacturing, and sometimes comparison to predicate devices (PCR-based tests) to demonstrate equivalence or superiority. Regulators may treat first-of-kind CRISPR tests as novel devices requiring more scrutiny (possibly even full Premarket Approval, PMA, if classified as high risk). In the European Union, the new In Vitro Diagnostic Regulation (IVDR) has raised the bar for demonstrating clinical performance and requires ongoing post-market surveillance. CRISPR tests for infectious diseases or critical diagnostics would fall under higher-risk categories, meaning companies must invest in extensive trials and documentation. This *regulatory hurdle* is non-trivial and could slow the entry of CRISPR diagnostics into the clinical market [249,252,264].

From a commercial standpoint, the interest is enormous. The versatility of CRISPR has drawn startups, and now larger diagnostic companies are beginning to take notice. The potential for point-of-care use and cost savings is attractive. However, companies will focus on demonstrating clear advantages over PCR in real-world settings to drive adoption. Speed, ease, and cost-effectiveness will be key selling points. Additionally, the IP landscape could shape the market: core patents on Cas12/Cas13 diagnostic uses are held by Sherlock and Mammoth, which might constrain who can deploy certain CRISPR systems commercially. Licensing deals or patent pooling may be necessary for broader industry uptake [264].

The explicit focus on enhanced clinical translation, commercialization, and regulatory aspects signifies a critical recognition that scientific breakthroughs alone are insufficient for real-world impact. This strategic emphasis aims to bridge the “valley of death” between laboratory innovation and widespread patient benefit [265,266,267]. While CRISPR diagnostics show immense promise in research, their widespread clinical adoption depends on overcoming practical hurdles related to manufacturing, quality control, and regulatory approval. This holistic approach, which couples scientific excellence with robust engineering, rigorous clinical validation, and clear regulatory pathways, is necessary to ensure that the inherent advantages of CRISPR diagnostics—speed, cost-effectiveness, and portability—are fully realized in healthcare systems globally. This ultimately leads to a tangible and sustainable impact on public health [268,269].

In recent years, we have seen the growing impact of CRISPR diagnostics, proven in principle and in pilot programs, with the first products in the pipeline, but not yet commonplace in hospitals or supermarkets. The next few years will determine how quickly they gain regulatory approvals, clinician, and public confidence. If they do, we could indeed witness a paradigm shift where CRISPR-based tests become a cornerstone of diagnostics, complementing or even replacing many PCR-based tests due to their combination of accuracy, speed, and accessibility [92].

## 8. Conclusions

CRISPR diagnostics have rapidly emerged as a transformative technology, offering unparalleled precision, speed, and versatility in detecting nucleic acids and other biomarkers across a broad spectrum of applications, including infectious diseases, cancer, and genetic disorders. Their inherent advantages, particularly in terms of portability, cost-effectiveness, and the potential for visual readouts, position them as ideal candidates for point-of-care applications and decentralized testing, addressing critical needs in diverse global health settings. While the field has made remarkable strides, challenges related to achieving consistent ultra-high sensitivity without pre-amplification, ensuring absolute specificity in complex biological matrices, overcoming PAM dependency, minimizing background noise, and simplifying upstream sample preparation steps persist [252,265,270]. Furthermore, navigating the complex landscape of clinical validation, standardization, and regulatory approval remains a significant hurdle for widespread adoption and commercialization.

The future trajectory of CRISPR diagnostics is characterized by a concerted and multidisciplinary effort to address these limitations. The integration of artificial intelligence promises to revolutionize assay design, data analysis, and biomarker discovery, leading to more intelligent and autonomous diagnostic solutions. Simultaneously, the development of universal CRISPR platforms, designed with modularity and broad applicability in mind, aims to create “plug-and-play” molecular diagnostic tools that can be rapidly deployed against emerging threats or for new biomarker discoveries. Ultimately, the success of CRISPR diagnostics hinges on bridging the gap between scientific innovation and practical implementation, ensuring that these powerful tools can move from the research bench to widespread clinical use, fundamentally reshaping disease diagnosis, surveillance, and personalized medicine on a global scale.

## Figures and Tables

**Table 1:** Overview of CRISPR-Cas Systems in Diagnostics.

Cas-Protein (Type)	Target	Characteristic Cleavage Mechanism	PAM Requirement	Key Diagnostic Feature
**Cas9 (Type II)**	DNA (dsDNA)	Cis-cleavage (double-strand breaks)	Yes (NGG)	Precise gene editing; Mutation detection
**Cas12a (Type V)**	DNA (dsDNA)	Trans-cleavage (ssDNA collateral)	Yes	High sensitivity via signal amplification
**Cas13a (Type VI)**	RNA (ssRNA)	Trans-cleavage (ssRNA collateral)	No (PFS)	Direct RNA detection
**Cas12b (Type V)**	DNA (dsDNA)	Trans-cleavage (ssDNA collateral)	Yes	One-pot/one-step assays
**Cas14a (Type V)**	DNA (ssDNA)	Trans-cleavage (ssDNA collateral)	No	PAM-free targeting for SNPs
**Cas10 (Type III)**	RNA (ssRNA)	Cyclic Oligoadenylate (cOA); indirect reporter cleavage	No	Orthogonal signaling pathway
**Cas3 (Type I)**	DNA (dsDNA)	Processive degradation (helicase + DNase)	Yes	Quantitative DNA degradation

**Table 2:** Representative Applications of CRISPR Diagnostics by Human Health and Disease Control.

Disease Area	Target/Pathogen/Biomarker	Protein	Techniques	Key Metrics (e.g., LOD, Sensitivity/Specificity, Time)
**Infectious Diseases**				
Bacterial	*Mycobacterium tuberculosis* DNA	Cas12a	RPA	LOD: 10 copies/reaction [18,19,20,21,22]
Bacterial	*Staphylococcus aureus* DNA	Cas12a	Impedimetric Biosensor	Ultra-sensitive detection [23,24,25,26,27,28]
Bacterial	*Klebsiella pneumoniae*	Cas12a	RPA	Rapid, highly specific [29,30,31,32]
Bacterial	*Salmonella* spp.	Cas12a/Cas12b	RPA/LAMP	Rapid, sensitive, visual [33,34,35,36,37]
Bacterial	*Brucella* spp. (BCSP31)	Cas12a	RPA	LOD: 10 copies/reaction (16.6 aM); Ultra-sensitive, accurate [38]
Viral	SARS-CoV-2 RNA	Cas12a/Cas13a	RT-RPA/ Smartphone	Time: ~20 min; Sensitivity: 0.5 copies/µL [39,40,41]
Viral	Human Adenoviruses (HAdV)	Cas12a	MCDA	Time: ~1 h; LOD: 1.92 copies/µL [42]
Viral	Human Papillomavirus (HPV)	Cas12a/Cas9	Amplification-free	Sensitive, amplification-free [18,43,44,45,46,47]
Viral	Infectious Bursal Disease Virus (IBDV)	Cas13a	RT-RPA/LFD	LOD: 5 aM (3 IBDV-RNA molecules); Superior sensitivity [48,49]
Viral	Monkeypox Virus (MPXV)	Cas12b	LAMP	Time: ~40 min; LOD: 6.5 copies/reaction; 100% sensitivity/specificity
Viral	Dengue Virus (DENV)	Cas13a	RAA	LOD: 10^−3^ copies·mL^−1^; 95.8% sensitivity/96.6% specificity [50,51,52,53,54,55,56,57]
Parasitic	*Anaplasma marginale*	Cas12a	RPA/Lateral Flow	LOD: 10^−2^ DNA copies/µL; Sensitive, user-friendly
Parasitic	*Toxoplasma gondii*	Cas12b	LAMP	Rapid, visual, accessible [58,59,60]
Plant Health	Tomato Leaf Curl Karnataka Virus (ToLCKV)	Cas12a	None (amplification-free)	10-fold lower detection than PCR (0.1 ng) [61]
**Cancer Diagnostics**				
Biomarker	Cancer-associated miRNAs	Cas12a/Cas13a	RT-RCA	LOD: 0.5 fM; 4–11x higher sensitivity [62,63,64,65,66]
Biomarker	BRAF V600E mutation	Cas12a	RPA	LOD: 2%; Time: 75 min [67,68]
Biomarker	Prostate Cancer Associated 3 (PCA3)	Cas12a	MIRA	Specificity: 83.3%; Time: 40 min [69]
**Genetic Disorders**				
Mutation	FBN1 variant (Marfan syndrome)	N/A	Whole Genome Sequencing	Identification of novel variant [70]
Disease Modeling	Duchenne Muscular Dystrophy	Cas9	Gene Editing	Rat model for cardiac function/pathology [71,72]
Disease Modeling	Galactosemia	Cas9	Gene Editing	Mouse model for lung injury [73]

**Table 3:** Comparative Analysis of CRISPR Diagnostic Assay Features and Performance.

Assay Name/Type (or Target)	CRISPR-Cas	Amplification	Detection Method	Limit of Detection (LOD)	Turnaround Time	Sensitivity/Specificity (if Available)	Key Advantage(s) Highlighted
SHERLOCK (IBDV)	Cas13a	RT-RPA	Lateral Flow (Visual)	5 aM	~1 h	Superior sensitivity to RT-qPCR	Ultrasensitive, POC
PalmCS (GBS)	Cas13a	RPA	Fluorescence/Smartphone	20 copies/reaction	~20 min	97.5% sensitivity/100% specificity	Portable, rapid, on-site
HAdV-MCDA-CRISPR (HAdV)	Cas12a	MCDA	Fluorescence (Visual)	1.92 copies/µL	~1 h	78/80 positive/48/48 negative	Rapid, highly specific
RPA-CRISPR/Cas12a (Brucella)	Cas12a	RPA	Fluorescence (Visual)	10 copies/reaction (16.6 aM)	~40 min	High sensitivity/Excellent specificity	Rapid, ultra-sensitive, accurate
RPA-CRISPR/Cas12a/Cas13a (SARS-CoV-2)	Cas12a/Cas13a	RT-RPA	Fluorescence/Smartphone	0.5 copies/µL	~20 min	High sensitivity	Ultrasensitive, portable, extraction-free
LAMP-CRISPR/Cas12b (MPXV)	Cas12b	LAMP	Fluorescence/Naked-eye	6.5 copies/reaction	~40 min	100% sensitivity/100% specificity	One-step, visual, accessible
MIRA-CRISPR/Cas12a (Prostate Cancer)	Cas12a	MIRA	Naked-eye (UV light)	0.01 ng/µL	~40 min	83.3% specificity (for PCA3)	Noninvasive, ambient temperature

**Table 4:** Comparative Analysis of CRISPR Diagnostic Platforms vs. PCR and NGS (2023–2025).

Characteristic	CRISPR-Based Assays	PCR (qPCR)—Gold Standard	Next-Generation Sequencing (NGS)
**Sensitivity (LoD)**	With amplification, attomolar-range LoD (as low as ~1–5 copies per reaction), comparable to qPCR. Without amplification, higher LoD (10^3^–10^6^ copies), though droplet digital CRISPR improves this.	~1–5 copies per reaction detectable (typical qPCR LoD). Very high analytical sensitivity for targeted assays. Digital PCR can reach single-copy detection.	Typically requires >10^3^ copies for confident detection unless target is amplified. Can detect variants down to ~1% allele frequency in mixtures, but not used for ultra-low copy *single-target* detection without pre-amplification.
**Specificity**	Extremely high—sequence-specific recognition by crRNA yields virtually no signal for off-target sequences. Capable of single-nucleotide discrimination with carefully designed guides (e.g., differentiating viral strains with 100% specificity). Low false-positive rates observed in studies.	High—determined by primer/probe design. Generally very specific, but can exhibit off-target amplification or primer-dimer artifacts if poorly designed. Single-base differences often require specialized probes or primers.	Highest—sequences are directly read out, so any off-target amplification can be identified by its sequence. NGS can distinguish every mutation in the target region. False positives mainly come from index misassignment or contamination, not mis-recognition of sequence.
**Time to Result**	Rapid—~30 to 60 min for most assays (including an isothermal amplification if needed). One-pot CRISPR tests can yield results in <30 min in some cases. Minimal sample prep; works at constant temperature.	Moderate—~1.5 to 2 h typical for a qPCR workflow (including sample prep and ~40 PCR cycles). Some rapid PCR systems ~30 min, but standard practice is longer. Requires thermal cycling.	Slow—hours to days. Library prep alone can take 3–10 h; sequencing run spans hours; plus, data analysis. Not suitable for urgent same-day answers in most cases.
**Cost per Test**	Low—Material cost on the order of USD 1 or less per test. Little to no capital equipment needed (simple incubator and reader or even lateral flow strips). Ideal for low-resource settings.	Moderate—Reagents a few dollars per test; however, requires expensive machines and trained personnel, which add to cost in lab settings. Economical at scale in centralized labs, but cost increases for point-of-care deployments due to equipment.	High—Sequencers are expensive; per-sample cost can range from USD 50 up to hundreds (depending on throughput and depth). Not cost-effective for single-target testing; mainly justified when a wealth of information is needed from each sample.
**Scalability & Throughput**	*Distributed scalability*: Can be deployed in many locations (decentralized testing). In the lab, parallel processing is improving (96-well formats, microfluidics), but it has traditionally lower throughput than PCR. Manual steps (pipetting, etc.) can limit high-throughput automation. New droplet CRISPR systems offer higher throughput potential.	*High throughput in central labs*: Easily run hundreds of samples on multi-well plates with automation. Scales well for large batch testing (e.g., mass screening). However, each test is individual (one sample, one result), and requires lab infrastructure.	*Massively parallel*, but in batch mode: Can sequence hundreds of samples and/or hundreds of targets in one run. High throughput for multiplexed analysis, but not in real-time—samples queue for batched runs. Scaling up means running more sequencers and handling big data.
**Multiplexing (Targets per Test)**	Moderate and evolving—small panels (2–5 targets) demonstrated by using multiple Cas enzymes or fluorescent reporters. Advanced methods (e.g., CARMEN droplet array) can test dozens of targets, but not yet routine. Generally, current CRISPR diagnostics are one target per assay, with multiplexing in R&D.	Limited—typically 4–6 targets in a single qPCR (due to distinct fluorescent channels). For more targets, run multiple reactions or use specialized multiplex PCR chemistries. Practical for small panels, but not scalable to dozens of targets in one reaction.	Very high—virtually unlimited targets (e.g., pathogen genome or a panel of 100+ genes) can be assessed simultaneously because each sequenced molecule is effectively an independent detection. Ideal for broad panels, though not needed for simple tests.
**Ease of Use and Equipment**	Designed for point-of-care: minimal equipment (often no electric devices for lateral flow readouts), simple protocol (mix and incubate). Can be performed by minimally trained users, even outside lab environments. Good for field use (e.g., farms, remote clinics).	Requires laboratory setup: precision thermal cycler, controlled environment to avoid contamination, and skilled technicians (especially for interpreting and troubleshooting). Not easily portable, though some cartridge-based PCR devices exist for near-patient use.	Requires advanced lab facilities: DNA sequencers, clean rooms for library prep, and bioinformatics for analysis. Only trained personnel in specialized labs can perform NGS. Not feasible outside a laboratory setting.
**Regulatory Status (as of 2025)**	Emerging—Received EUAs in the U.S. for COVID-19 tests. No fully FDA-approved clinical CRISPR tests yet; undergoing trials. Subject to rigorous IVD regulations (FDA, EU IVDR) which demand extensive validation. Moving toward approvals for infectious diseases and others in coming years.	Established—Decades of use; PCR-based tests are routinely approved by regulatory agencies for diagnostics. Well-defined pathways for clearance (510(k) or PMA), and many FDA-approved PCR kits exist for various diseases. Widely accepted standard in clinical labs.	Established—Used in specialized diagnostics (e.g., comprehensive genomic profiling). Some NGS-based IVDs have FDA approval (especially in oncology). Heavily regulated and used mostly in high-complexity labs. Not typically used for simple infectious disease diagnostics due to overkill nature, but increasingly approved for complex testing (e.g., metagenomic infection diagnosis, cancer gene panels).

## Data Availability

No new data were created or analyzed in this study.

## Abbreviations

AI	Artificial Intelligence
ARDS	Acute Respiratory Distress Syndrome
Cas	CRISPR-associated
CLIA	Clinical Laboratory Improvement Amendments
cOA	Cyclic Oligoadenylate
CRISPR	Clustered Regularly Interspaced Short Palindromic Repeats
crRNA	CRISPR RNA
dCas9	Catalytically Inactive version of Cas9
DENV	Dengue Virus
DSB	Double-Strand Break
EUA	Emergency Use Authorization
EV	Extracellular Vesicle
FDA	Food and Drug Administration
GBS	Group B Streptococcus
HAdV	Human Adenoviruses
HPV	Human Papillomavirus
IBDV	Infectious Bursal Disease Virus
IP	Intellectual Property
IVD	In Vitro Diagnostic
IVDR	In Vitro Diagnostic Regulation
LAMP	Loop-Mediated Isothermal Amplification
LFA	Lateral Flow Assay
LOD	Limit of Detection
MCDA	Multiple Cross Displacement Amplification
miRNA	microRNA
MPXV	Monkeypox Virus
PAM	Protospacer Adjacent Motif
PCA3	Prostate Cancer Associated 3
PFS	Protospacer Flanking Site
PMA	Premarket Approval
POC	Point-of-Care
PSA	Prostate-Specific Antigen
RAA	Recombinase Aided Amplification
RPA	Recombinase Polymerase Amplification
SERS	Surface-Enhanced Raman Spectroscopy
SFTSV	Severe Fever with Thrombocytopenia Syndrome Virus
sgRNA	Single Guide RNA
SNP	Single-Nucleotide Polymorphism
ssDNA	Single-Stranded DNA
ssRNA	Single-Stranded RNA
TB	Mycobacterium tuberculosis
ToLCKV	Tomato Leaf Curl Karnataka Virus
